# Retraction Note: Calcium-dependent cytosolic phospholipase A2 activation is implicated in neuroinflammation and oxidative stress associated with ApoE4

**DOI:** 10.1186/s13024-022-00519-x

**Published:** 2022-02-04

**Authors:** Shaowei Wang, Boyang Li, Victoria Solomon, Alfred Fonteh, Stanley I. Rapoport, David A. Bennett, Zoe Arvanitakis, Helena C. Chui, Carol Miller, Patrick M. Sullivan, Hoau-Yan Wang, Hussein N. Yassine

**Affiliations:** 1 Departments of Medicine and Neurology, Keck School of Medicine, University of Southern California, Los Angeles, CA USA; 2 Huntington Medical Research Institutes, Pasadena, CA USA; 3 National Institute on Alcohol Abuse and Alcoholism, Bethesda, MD USA; 4 Rush Alzheimer’s Disease Center, Rush University Medical Center, Chicago, IL USA; 5 Department of Medicine, Duke University Medical Center, Durham Veterans Health Administration Medical Center’s Geriatric Research, Education and Clinical Center, Durham, NC USA; 6 The City University of New York School of Medicine, New York, NY USA; 7 Graduate School of The City University of New York, New York, USA

**Retraction Note: Mol Neurodegener 16, 26 (2021)**

**https://doi.org/10.1186/s13024-021-00438-3**

The authors have retracted this article because concerns have been raised regarding the data presented in Fig. 9. The authors are collecting new data and intend to submit a new manuscript for peer review in due course.

Authors Shaowei Wang, Boyang Li, Victoria Solomon, Alfred Fonteh, Stanley I. Rapoport, David A. Bennett, Zoe Arvanitakis, Helena C. Chui, Patrick M. Sullivan, Hoau-Yan Wang & Hussein N. Yassine agree to this retraction.

Author Carol Miller has not responded to any correspondence from the editor or publisher about this retraction.

